# From Genotype to Phenotype: Expanding the Clinical Spectrum of *CACNA1A* Variants in the Era of Next Generation Sequencing

**DOI:** 10.3389/fneur.2021.639994

**Published:** 2021-03-02

**Authors:** Elisabetta Indelicato, Sylvia Boesch

**Affiliations:** Center for Rare Movement Disorders Innsbruck, Department of Neurology, Medical University of Innsbruck, Innsbruck, Austria

**Keywords:** *CACNA1A*, calcium channels, developmental delay, epileptic encephalopathy, dystonia, next generation sequencing, *de novo* mutation, psychiatric manifestations

## Abstract

Ion channel dysfunction is a key pathological substrate of episodic neurological disorders. A classical gene associated to paroxysmal movement disorders is *CACNA1A*, which codes for the pore-forming subunit of the neuronal calcium channel P/Q. Non-polyglutamine *CACNA1A* variants underlie familial hemiplegic ataxia type 1 (FHM1) and episodic ataxia type 2 (EA2). Classical paroxysmal manifestations of FHM1 are migraine attacks preceded by motor aura consisting of hemiparesis, aphasia, and disturbances of consciousness until coma. Patients with EA2 suffer of recurrent episodes of vertigo, unbalance, diplopia, and vomiting. Beyond these typical presentations, several reports highlighted manifold clinical features associated with P/Q channelopathies, from chronic progressive cerebellar ataxia to epilepsy and psychiatric disturbances. These manifestations may often outlast the burden of classical episodic symptoms leading to pitfalls in the diagnostic work-up. Lately, the spreading of next generation sequencing techniques linked *de novo CACNA1A* variants to an even broader phenotypic spectrum including early developmental delay, autism spectrum disorders, epileptic encephalopathy, and early onset paroxysmal dystonia. The age-dependency represents a striking new aspect of these phenotypes und highlights a pivotal role for P/Q channels in the development of the central nervous system in a defined time window. While several reviews addressed the clinical presentation and treatment of FHM1 and EA2, an overview of the newly described age-dependent manifestations is lacking. In this Mini-Review we present a clinical update, delineate genotype-phenotype correlations as well as summarize evidence on the pathophysiological mechanisms underlying the expanded phenotype associated with *CACNA1A* variants.

## Introduction

**Figure 1:**
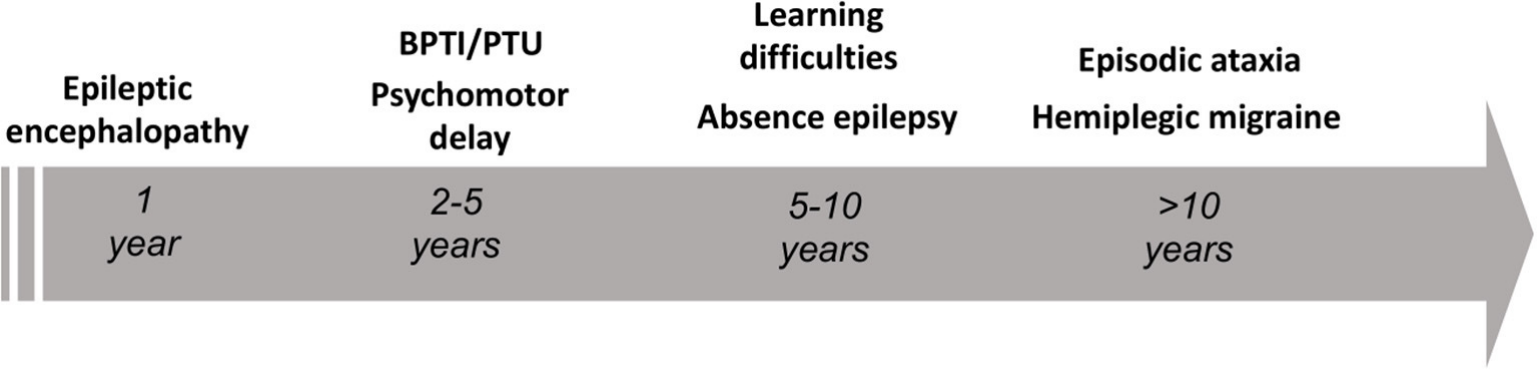
Schematic representation of the age-dependent phenotypes encountered in the setting of pathogenic *CACNA1A* variants. BPTI, benign paroxysmal torticollis of the infancy; PTU, paroxysmal tonic upward gaze.

The gene *CACNA1A* encodes the α1A pore-forming subunit of the neuronal calcium channel P/Q (1). The first association of *CACNA1A* with human diseases dates back to 1996, as Ophoff et al. (2) described its mutations in two allelic episodic neurological disorders, familial hemiplegic migraine type 1 (FHM1) and episodic ataxia type 2 (EA2). FHM1 presents with episodes of migraine with motor symptoms during the aura (3), while EA2 features attacks of paroxysmal gait disturbances, dysarthria and diplopia (3, 4). Already in 1997, a third disease, the spinocerebellar ataxia type 6 (SCA6), was mapped to *CACNA1A* locus (5). Differently from paroxysmal *CACNA1A* disorders, SCA6 is a late-onset disease manifesting as progressive isolated cerebellar ataxia (6). Far from being the first anomaly in the paradigm, our knowledge on *CACNA1A* disorders continued expanding over the past 25 years, revealing an unexpected variability both from a genotypic and phenotypic point of view. Established manifestations of *CACNA1A* variants comprehend neuropsychiatric disorders (7, 8), paroxysmal dystonia (9–11), epilepsy (12, 13), as well as complex phenotypes characterized by a various combination of early developmental delay and epileptic encephalopathy (14–16). Nowadays, an emerging hypothesis highlights an age-dependency of *CACNA1A* phenotypes (see Figure 1).

The present review is an update on the expanded disease spectrum associated to *CACNA1A* variants. After a brief introduction on molecular genetics of P/Q channels, we review the related literature, delineate genotype-phenotype correlations and highlight translational aspects. Our aim is to offer a guide for the clinician as to when to apply a *CACNA1A* testing beyond the typical FHM1/EA2 phenotype.

## Methods

Pertinent literature was retrieved through a Medline search using the following mesh terms in various combinations: *CACNA1A*, P/Q calcium channels, epilepsy, developmental delay, mental retardation/intellectual disability, epileptic encephalopathies, dystonia, and animal models. Furthermore, we considered relevant references from selected papers and consulted publicly accessible databases (ClinVar, Genecards, Uniprot).

## P/Q Calcium Channels: Pathophysiology and Molecular Genetics

**Figure 2:**
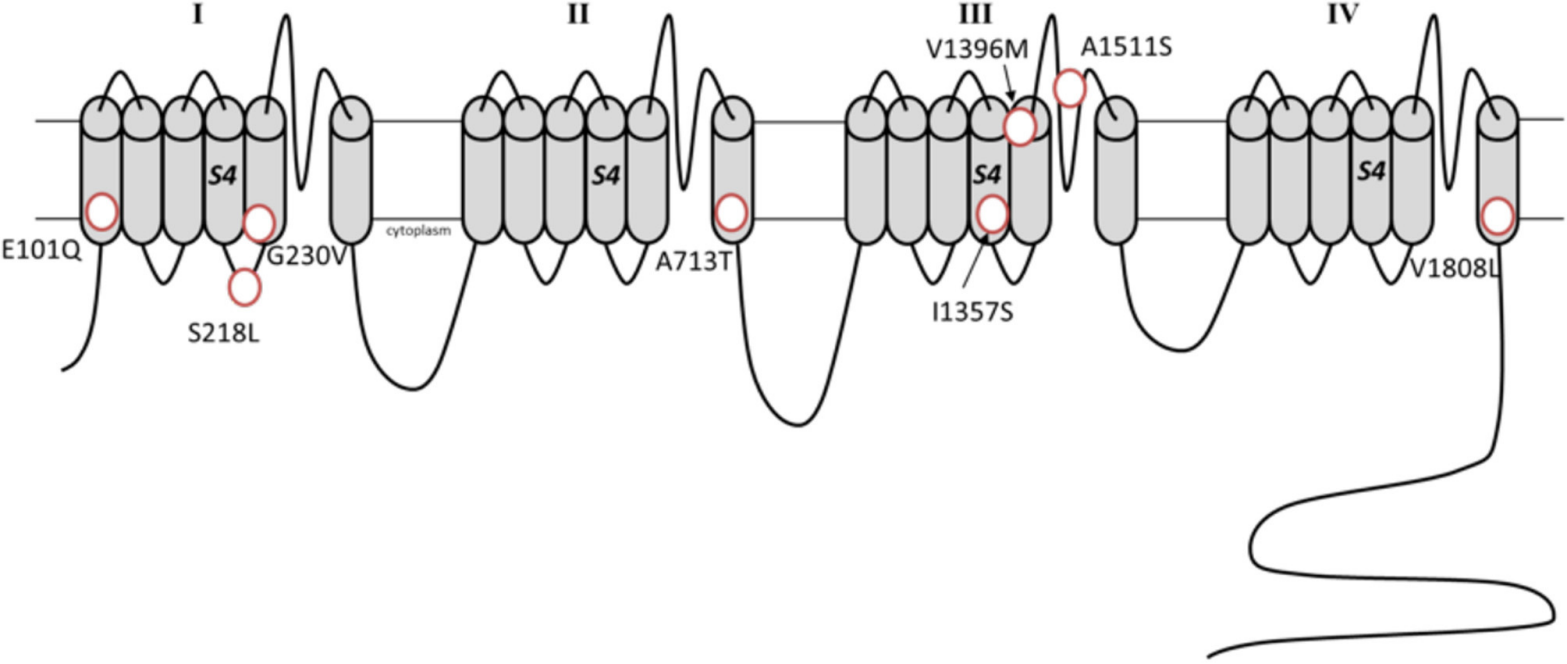
Schematic representation of the α1A subunit with the *de novo* missense variants associated with developmental epileptic encephalopathies (Transcript variant 2, NM_023035.2).

The P/Q calcium channels are voltage-gated channels consisting of a pore-forming α-subunit connected with further regulatory subunits (α2δ dimer and one or more β subunits) (17). The α-subunit determines the ion-specificity as well as the kinetic properties of the channels (18). Figure 2 offers a schematic representation of the unfolded α1A-subunit coded by *CACNA1A*. The P/Q channels are ubiquitous at central synapses and particularly abundant in cerebellar granules and Purkinje cells (18). From the presynaptic terminals P/Q channels orchestrate neurotransmitter release (19). Notably, calcium currents are not only involved in the electrochemical coupling during neurotransmission. Indeed, calcium acts also as a second messenger in the cells (20), being involved in the development of synaptic plasticity (21–23) and further regulatory processes in the central nervous system (24–26).

The *CACNA1A* gene is located on chromosome 19. It contains 47 exons, many of which undergo a canonical alternative splicing that results in myriad isoforms with different functional properties and regional expression patterns (27). *CACNA1A* scores among the 2% most intolerant genes of the human genome (28). Particularly the transmembrane region shows paucity of variations (29). The spectrum of disease associated *CACNA1A* variants spans from point mutations (missense and nonsense) to short indels and larger deletions (30–32). Pathogenic variants can also affect intronic sequences leading to translational frameshift (33). Several disease-associated missense variants lead to substitution of positively charged arginine in the segment S4 (29, 34–36). An updated graphical overview of the known mutations in CACNA1A can be found in (37) and (38). Irrespective of the mutation type, *CACNA1A* diseases shown an autosomal dominant pattern of inheritance. Concerning genotype-phenotype correlations, FHM1 is generally associated with missense gain-of-function mutations (39), while EA2 usually results from variants with loss-of-function effect, including truncating mutations and small deletions (40). Although sharing some paroxysmal symptoms at the beginning of disease, SCA6 is not directly affecting the channel kinetics as its allelic disorders. SCA6 is caused by an expanded CAG repeat coding for a polyglutamine tract (41). Recent research revealed that *CACNA1A* contains an “internal ribosome entry site,” which initiates the translation of a second peptide, referred to as α1ACT. This second product consists of a C-terminal fragment containing the expanded polyglutamine tract causing SCA6 (42). The peptide α1ACT is not involved in channel formation, but acts as a transcription factor and regulates the expression of key genes involved in synaptic formation, neurogenesis, and cell adhesion (42). The functionality of α1ACT in a specific time-window drives the maturation of the Purkinje cells in the early development (43).

## The Expanded Phenotype of *CACNA1A* Variants

### Classical Manifestation: Familial Hemiplegic Migraine Type 1 and Episodic Ataxia Type 2

#### Clinical Presentation and Genotype-Phenotype Correlation

FHM1 is a monogenic form of migraine characterized by the occurrence of motor deficits during the aura (44). The classical motor aura in FHM1 consists of a transient hemiparesis, but a variety of other neurological symptoms can be present, such as dysphasia, sensory loss, visual disturbance and vertigo (3). These deficits often outlast the associated headache and are extended for hours to days (3). The frequency of attacks is very variable and may range from 1 to 2 in an entire lifespan to several per month (39, 45). FHM1 is usually underlain by missense *CACNA1A* mutation with gain of function effect on calcium currents (2). The onset is usually in the first or second decade (3).

EA2 is the most common form of episodic ataxia (2). EA2 manifests with attacks of ataxia with nausea and vomiting. Attacks typically last minutes to days and can be associated with diplopia, dysarthria, tinnitus, dystonia, hemiplegia, and headache, also migraine headaches (4, 46). The frequency of attacks is very variable. Attacks can range from once or twice a year to several per day (47). Stress, caffeine, alcohol, exertion, fever, heat, and phenytoin can trigger attacks (48). The typical onset is in early adolescence or childhood (47). Truncating *CACNA1A* mutations with loss of function effect are the typical genotypic finding in EA2.

In FHM1 and EA2 symptoms may often overlap (13, 40, 49). Even if several patients present with isolated paroxysmal symptoms, the majority will eventually develop mild to moderate chronic cerebellar signs in the course of the disease (4, 39).

### Developmental Delay

#### Clinical Presentation

Episodes of hemiplegic migraine or paroxysmal ataxia typically arise in the first two decades of life. Though, a focused clinical history may reveal earlier symptoms in a substantial proportion of patients. Indeed, the parents of older children and adolescents with EA2 and FHM1 can often recall some delay in the acquisition of the early milestones, for instance delayed walking or speaking (7, 45). Learning deficits as well as behavioral abnormalities consistent with attention-deficit hyperactivity disorder or autistic spectrum disorders can dominate the clinical picture in schoolchildren (4, 14, 34, 35, 50, 51). Although natural history data are lacking, the available literature and our clinical experience suggests that a compensation of apparently marked deficits can take place in the disease course. Adult patients who suffered from early delay or required special education can still conduct a normal working and social life (45, 52). Reports of rapid cognitive deterioration starting in adulthood are exceptional (53). In the past decade, the increasing availability of next generation sequencing (NGS) techniques added *de novo* missense *CACNA1A* mutations to the genetic background of intellectual disability. Indeed, several studies applying NGS discovered pathogenic *CACNA1A* variants in children with severe psychomotor delay born from healthy parents (54–56). Intellectual disability was the prominent symptom in these cases. Although motor examination can reveal a marked hypotonia (54, 55), clear signs of ataxia may become apparent only later in small children. In several cases, intellectual disability occurs along with therapy-resistant epilepsy, leading to severe clinical pictures in the spectrum of developmental epileptic encephalopathies (see next paragraphs).

#### Pathophysiology

The pathophysiological basis of intellectual disability due to pathogenic *CACNA1A* variants is largely unexplored. In a single animal study, the role of P/Q channels in circuitry underlying cognition and memory was investigated by means of a conditional murine model with selective knock-out of *CACNA1A* in the forebrain. The loss of P/Q currents in the neocortex and hippocampus led to marked deficits in memory, spatial learning and in increased exploratory behavior in the mouse (57). As mentioned above, both P/Q calcium currents and the transcription factor α1ACT have an established role in the early maturation of the cerebellum. Perturbations in the developing cerebellum underlid by P/Q channel and α1ACT dysfunction may contribute to occurrence of neuropsychiatric disorders early in life by altering the cerebellar tuning on cognitive cortical networks (58). This hypothesis is in line with the notion of a “cerebellar cognitive-affective syndrome” (59), which can arise independently from an ataxic motor phenotype in the setting of cerebellar disorders of various etiologies.

In the last decade, a syndrome with severe developmental delay and dysmorphic features has been described in association with microdeletion of chromosome 19 (19p13.13 or 19p13.2) containing also the *CACNA1A* gene (60–62). These findings corroborate on one hand the association between *CACNA1A* and neurodevelopmental disorders, on the other underlie the likely relevant contribute of multiple genetic factors—along with single gene variants—as background of such severe phenotypes.

### Epilepsy in Classical Paroxysmal *CACNA1A* Disorders

#### Clinical Presentation and Genotype-Phenotype Correlation

An association between epilepsy and *CACNA1A* disorders has been postulated early (12) and then corroborated by several independent reports describing co-occurrence of epileptic features in *CACNA1A* pedigrees [reviewed in (63)]. Epileptic features display some genotype-specificity. Indeed, seizures and epileptiform activity in EEG are a recurrent finding in EA2 (12, 14, 35, 38, 40, 47, 64–67). Epileptic features are observed in childhood and adolescence, often before onset of episodic ataxia (31, 38, 40, 64, 68). Absences and 3 Hz Spike-Wave discharges are typical findings (13, 14, 38, 65, 67). Conversely, in pedigrees with FHM1 seizures mostly occur concomitantly to hemiplegic attacks (69–74) and independent epileptic manifestations are unusual (51, 75–77).

#### Pathophysiology

Beyond the clinical field, several lines of evidence support the association between a loss-of-function of P/Q channels and generalized epilepsy. Notably, mutant mice bearing loss-of-function *CACNA1A* variants represent established models of absence epilepsy (78). In these models (Tottering, Leaner, Rocker) absence-like seizures go along with an ataxic motor phenotype (79–82). Mice with *CACNA1A* null mutation develop a similar phenotype with absence epilepsy and progressive neurological deficits before dying in the age of 3–4 weeks (83).

From a pathophysiological point of view, the EEG changes driving absence depend on an abnormal synchronization of thalamocortical circuitry (80, 84). Several studies showed that a loss-of-function of P/Q channels results in increased T-type current in the thalamocortical network (78, 79). T-type calcium current are directly involved in the generation 3 Hz Spike-Wave rhythmicity and overexpression of T-type currents in mice is sufficient to induce absence epilepsy (85). Interestingly, a study in a conditional murine model showed that absence epilepsy occurs also when P/Q loss in limited to Purkinje cells (86). This notion suggests a relevant regulatory role for early cerebellar inputs in thalamocortical circuitry. Indeed, a reduced firing in Purkinje cells with knock-out of P/Q currents decreases inhibition on deep cerebellar nuclei and in turn leads to increasing output of cerebellar projections to the thalamus.

### Epileptic Encephalopathy

#### Clinical Presentation and Genotype-Phenotype Correlation

Latest clinical studies in early onset epileptic encephalopathies suggest that the mechanisms subserving epileptogenesis in the setting of pathogenic *CACNA1A* variants is likely more complex than anticipated. Epileptic encephalopathies are devastating neurological disorders characterized by early onset of multiple seizure types, accompanied by psychomotor regression and a variety of focal neurological signs including ataxia and cerebral palsy (87). The severe and frequent epileptic activity is believed to be the pivotal trigger for further psychomotor deterioration. Originally, epileptic encephalopathies were considered to result mostly from a symptomatic etiology, such as perinatal hypoxic-ischemic insults. The discovery of mutations in the sodium channel gene *SCN1A* in the Dravet syndrome in 2001 paved the way to the exploration of their genetic background (88). Since then, cumulative research highlighted how *de novo* mutations in channel genes are a recurrent cause of epileptic encephalopathies and recently revealed a role also for *CACNA1A* variants (15). Indeed, *CACNA1A* variants have been found in the setting of severe epileptic syndromes including Ohtahara syndrome, epilepsy of infancy with migrating focal seizures and Lennox-Gastaut syndrome.

A phenotype compatible with epileptic encephalopathy has first been described in association with deletions and truncating mutations in *CACNA1A* (14). Afterwards, the application of NGS techniques in large collectives detected *de novo* missense *CACNA1A* variants in this setting (15, 89–92). The observed phenotype in the affected individuals reflected the typical course of an epileptic encephalopathy with the beginning of multiple seizure types in the first days of life as well as various degrees of intellectual disability. The pathophysiological mechanisms linking such a set of functionally heterogeneous genotypes with this devastating clinical picture are unclear (93). Detected variants include both newly described and well-known ones, such as the S218L, which is typically associated with a severe form of FHM1 (70). Despite current evidence is limited to few studies, some mutations have been already recurrently observed [e.g., A713T (15, 94, 95) and E101Q (15, 91)].

#### Pathophysiology

A recent study investigated the functional effect of the variants associated with epileptic encephalopathies (96). For example, the variants G239V and I1357S resulted in altered protein trafficking with accumulation in intracellular inclusions in HEK293 cells, a finding compatible with a dominant negative effect. The variants A713T and V1396M, showed a gain-of-function effect, which appeared more profound compared to that of classical gain-of-function mutations seen in FHM1 (96). Notably, the variant S218L, which underlies both a severe FHM1 form and epileptic encephalopathy, has also a well-established more deleterious effect on current activation and inactivation (97, 98). These notions suggest that the absolute degree of channel dysfunction, more than the specific effect on currents kinetics, drives severe phenotypes. The missense mutations associated with developmental epileptic encephalopathies are situated in transmembrane segments, except for the variant S218L which localizes in a cytosolic region (see Figure 2). The transmembrane located variants likely affect the voltage-sensor or pore domains, while it remains speculative if the intracellular located S218L variant may additionally alter the binding with intracellular protein/intracellular signaling.

### Early Onset Dystonia

#### Clinical Presentation

Dystonia can occur in a number of primary cerebellar disorders including the autosomal-dominant spinocerebellar ataxias and several autosomal recessive ataxias. *CACNA1A* disorders may also feature dystonic symptoms, with some typical age-specific patterns. Several reports highlighted the occurrence of a periodic movement disorders such the benign paroxysmal torticollis of the infancy (BPTI) (9–11, 45, 99) and the paroxysmal tonic upward gaze (PTU) (10, 100, 101) in infants from *CACNA1A* pedigrees. Both conditions are rare and their occurrence is typically limited to the infancy. BPTI consists of transient episodes of cervical dystonia, which may be accompanied by vomiting, ataxia, pallor. The attacks usually remit spontaneously within 3–5 years of age (102). Infants with BPTI often have a positive family history for migraine. BPTI can later evolve in migraine or further migraine-related disorders and has been included in the third edition of the International Classification of Headache Disorders (44). Its “benign” course is an object of controversy, since a number of cases show a developmental delay (102). Notably, a recent study screening a larger group of infants with BPTI but without further neurological symptoms did not find *CACNA1A* variants (103). In general, a suspect for an underlying pathogenic *CACNA1A* variant is strong when BPTI arises in infants with positive family history for FHM/EA or presence of delayed milestones. PTU consists of episodes of sustained conjugate upward deviation of the eyes, with neck flexion and sometimes impaired coordination, lasting from minutes to hours (104). PTU was originally also believed to be a benign phenomenon, resolving without sequelae. Later reviews demonstrated that it may be associated to structural brain pathologies and predate a severe neurological phenotype with chronic ataxia, epilepsy and cognitive impairment (105, 106). Very recently, a multicentric study on 47 infants with *CACNA1A* variants revealed a high frequency of paroxysmal dystonia, especially of PTU (found in 47% of cases, while torticollis was evident in 19%) (107). In adult patients with EA2, dystonia, mostly cervical, can occur during paroxysmal manifestations (46, 108) or persist as chronic symptoms later in the disease course (49, 109–111). Pathophysiological background of paroxysmal dyskinesias has been investigated in tottering and rocker mice bearing loss-of-function *CACNA1A* mutations (112).

## Discussion

The advent of NGS techniques revolutionized our understanding of a relevant number of neurological diseases. Especially in the field of neurodevelopmental disorders, the identification of *de novo* pathogenic variants in ion channel genes opened a new window of opportunities for unraveling their pathophysiological mechanisms. Within these studies, *CACNA1A* variants also emerged as a relevant novel genetic etiology.

### When to Choose a Genetic Testing for *CACNA1A*

*CACNA1A* testing belongs to the standard assessment of hemiplegic migraine or episodic ataxia, especially in the setting of a positive family history or when chronic cerebellar signs are present (113).

**Figure 3:**
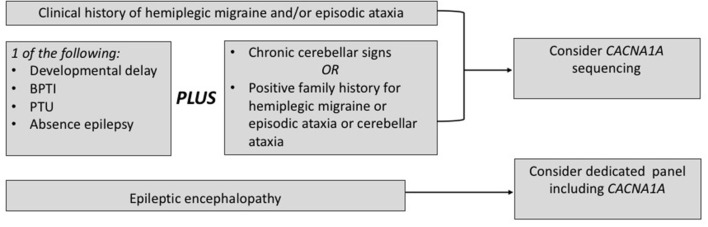
When to choose a genetic testing for CACNA1A. BPTI, benign paroxysmal torticollis of the infancy; PTU, paroxysmal tonic upward gaze.

Based on the current literature, *CACNA1A* sequencing should be considered also in the settings of early epilepsy and/or developmental delay when chronic cerebellar signs or a positive family history for FHM/EA are present. Concerning the chronic cerebellar signs, an isolated downbeat nystagmus may be a decisive clue (14). Similarly, a clinical picture with early onset paroxysmal dystonia, such as BPTI, plus cerebellar signs supports the application of *CACNA1A* sequencing, even in the presence of a negative family history. Furthermore, current evidence from the field of epileptic encephalopathies supports the application of diagnostic panels covering also *CACNA1A* in this setting. A flow chart in Figure 3 summarizes the present recommendations.

### Therapeutic Implications

The detection of a causal *CACNA1A* variant in the setting of a paroxysmal disorder bears relevant therapeutic consequences. Indeed, the classical paroxysmal manifestations such as hemiplegic migraine and episodic ataxia usually display a good to excellent response to the interval therapy with acetazolamide (40, 114). The exact mechanism underlying this therapeutic effect is unclear. Acetazolamide is a carbonic anhydrase inhibitor and induces a pH shift in liquor toward more acidic values. Changes in extracellular pH influence membrane conductance and may induce a stabilization of P/Q channel permeability (40). Second line compounds in non-responsive cases are 4-aminopyridine and flunarizine (114, 115). Generally, these therapies are not expected to improve chronic neurological symptoms, although their stabilization upon beginning of medical treatment can be occasionally reported (46, 116). A further exception is represented by improvement of down-beat nystagmus under 4-aminopyridine treatment (115).

Therapy-resistant seizures markedly contribute to developmental involution in epileptic encephalopathies and thus the timely initiation of an effective antiepileptic treatment represents a crucial issue. Since seizures are episodic manifestations *sensu strictu*, the question raises whether they may also improve upon acetazolamide as other paroxysmal symptoms. Acetazolamide has a known anticonvulsive effect, which also depends on the induction of an acidotic state (117). Because of its tolerance developing properties and side effects (electrolytes derangements, nephrolithiasis), acetazolamide is currently used mostly as add-on therapy in refractory epilepsies or in special situations, e.g., catamenial epilepsy (118). Interestingly, the established anticonvulsive drug topiramate also exhibits carbonic anhydrase inhibition properties and it has occasionally been applied with success in the treatment of FHM1/EA2 (45). In the largest reported series of *CACNA1A*-related epileptic encephalopathy, four out of six cases were upon treatment with acetazolamide or topiramate but information about the treatment response was not provided (15).

A detailed presentation of the treatment of manifestations associated with *CACNA1A* mutations is beyond the scope of this mini-review and for that we refer to the several reviews on the topic (40, 114).

### Open Questions for Future Research

Despite the expanding knowledge on molecular genetics and clinical presentation of *CACNA1A* variants, the lack of prospective data from large collectives limits our understanding of this channelopathy. For example, it remains to be elucidated if *CACNA1A*-related neurodevelopmental disorders represent separate entities or rather an age-dependent stage within an evolving phenotype. The responsivity of early developmental/epileptic features to the classical interval therapy remains also an open question for clinicians. Future clinical studies with a prospective design may clarify these issues. A further puzzling issue remains the coexistence of marked intrafamilial variability as well as phenotypic overlap across different mutation types. These issues limit the definition of a clear-cut genotype-phenotype correlation for the clinician. On the bench side, a deeper understanding of implications of *CACNA1A* dysfunction on cerebrocerebellar circuitries and local plasticity phenomena at the cellular membrane may help to conceive common therapeutic strategies targeting symptoms across different phenotypes (37).

## Author Contributions

EI and SB both contributed to conceive, write, and review the manuscript. All authors contributed to the article and approved the submitted version.

## Conflict of Interest

The authors declare that the research was conducted in the absence of any commercial or financial relationships that could be construed as a potential conflict of interest.
